# A framework for comparative study of databases and computational methods for arrhythmia detection from single-lead ECG

**DOI:** 10.1038/s41598-023-38532-9

**Published:** 2023-07-19

**Authors:** Elena Merdjanovska, Aleksandra Rashkovska

**Affiliations:** 1 Department of Communication Systems, Jožef Stefan Institute, 1000 Ljubljana, Slovenia; 2 Jožef Stefan International Postgraduate School, 1000 Ljubljana, Slovenia

**Keywords:** Biomedical engineering, Computer science, Cardiology

## Abstract

Arrhythmia detection from ECG is an important area of computational ECG analysis. However, although a large number of public ECG recordings are available, most research uses only few datasets, making it difficult to estimate the generalizability of the plethora of ECG classification methods. Furthermore, there is a large variability in the evaluation procedures, as well as lack of insight into whether they could successfully perform in a real-world setup. To address these problems, we propose an open-source, flexible and configurable ECG classification codebase—ECGDL, as one of the first efforts that includes 9 arrhythmia datasets, covering a large number of both morphological and rhythmic arrhythmias, as well as 4 deep neural networks, 4 segmentation techniques and 4 evaluation schemes. We perform a comparative analysis along these framework components to provide a comprehensive perspective into arrhythmia classification, focusing on single-lead ECG as the most recent trend in wireless ECG monitoring. ECGDL unifies the class information representation in datasets by creating a label dictionary. Furthermore, it includes a set of the best-performing deep learning approaches with varying signal segmentation techniques and network architectures. A novel evaluation scheme, inter-patient cross-validation, has also been proposed to perform fair evaluation and comparison of results.

## Introduction

Electrocardiogram (ECG) is the standard to obtain information about the electrical function of the heart and computational ECG analysis has been researched for over 60 years^1^. The development of automatic ECG-based heartbeat classification and arrhythmia detection methods represents a large portion of the research involving computational methods for ECG analysis^2^.

The research on this topic has involved some standard methods in the past, such as frequency analysis^3^, wavelet transform^4^ and template matching^5^. In recent years, the focus has started to shift towards machine learning methods^6^, with the majority of state-of-the-art arrhythmia detection studies now using deep learning techniques^7^. Convolutional neural networks (CNNs)^8^ are most commonly used for all learning tasks related to images and signals, as well as ECG. CNNs have been used for arrhythmia detection on a wide range of datasets, evaluation scenarios, as well as various target class groupings^9–11^. In addition, attention and long short-term memory (LSTM)^12^ mechanisms have successfully been employed for this task.

In order to be able to develop automatic ECG analysis methods, example databases are necessary. There are a lot of publicly available databases containing ECG recordings which are of high importance. Studies analyzing data available to everyone have a higher value because this allows for fair comparison and reproducibility of the results. Public ECG databases include ECG signals with different recording settings, ranging from the standard 12-lead resting ECG to monitoring with the Holter monitor. Usually, databases with a higher recording frequency and 12 or 15 leads are obtained with the standard resting ECG monitor^13,14^, while those with a few leads are obtained with the Holter monitor^15^. Additionally, the recordings have various duration, ranging from 10 s^16^ up to 24 h^17^, and different sampling frequencies, usually 250 Hz or higher. Most public ECG databases are available through public repositories, like PhysioNet^18^, Figshare^19^, Zenodo^20^ and IEEE Data Port^21^. The use of these databases is largely determined by the annotation types, and the additional signals and information they contain parallel to the ECG recordings. Moreover, in the last years, we have seen more and more large-scale arrhythmia datasets being published, like the PTB-XL database^14^.

Even though a large number of public ECG recordings is available, the largest portion of research uses only few datasets, most commonly the MIT-BIH Arrhythmia Database (MIT-BIH-AR)^15^. In addition, there is a large variability in the evaluation of the methods, as well as lack of insight into whether they could successfully perform in a real-world setup. The evaluation methods vary according to whether they include heartbeats from the same person in both the train and test datasets, as well as if the model is personalized for a patient or is a general one. This makes it very difficult to compare different models that use different evaluation strategies. Also, as mentioned, most of the methods are specialized for one dataset, which makes it difficult to estimate the generalization abilities of the models. An analysis of how proposed models could perform on data from a different institution, acquired with different measurement technology or containing different ECG leads, is lacking in such studies.

During the years, commercial closed-source software products for computational ECG analysis, which incorporate also diagnostic ECG classification techniques, have been developed. Older traditional software solutions, like Uni-G^22^ and Hanover^23^, are intended for implementation on ECG measuring devices. Modern ECG analysis cloud-based services, like Cardiomatics^24^ and Cardiologs^25^, incorporate more complex machine learning techniques, like deep learning. As opposed to commercial tools (with hidden implementations for the most part), open-source tools enable the advancement of research in the field. Making all data and code available ensures reproducibility in the computational sciences, which is one of the main scientific principles^26^. There are several open-source recent tools for computational ECG analysis, such as PALMS (Platform for Analysis and Labeling of Medical Time Series)^27^, as well as frameworks incorporating various machine learning methods for arrhythmia detection^28–30^. However, although these frameworks usually include multiple methods, they are limited to a specific evaluation procedure^28^ or dataset^30^.

A comprehensive evaluation of the best-performing methods for arrhythmia detection as a classification task, both on large-scale public databases, as well as on measurements from mobile ECG devices, is needed. We aim to address this problem by providing an open-source comprehensive benchmarking environment for arrhythmia detection focusing on deep learning techniques – ECGDL, avaliable at https://github.com/elenamer/ecg_classification_DL. ECGDL attempts to overcome the limitations of existing open-source ECG classification frameworks by covering a wide range of datasets, as well as fair evaluation procedures to assess the performance and generalization abilities of state-of-the-art models. The analyses will provide more insight into exactly how much the challenges brought by mobile ECG sensors influence the performance of existing methods. To address this, we focus on methods utilizing only one ECG lead, either from single-lead ECG sensors or by choosing one lead in multi-lead datasets.

## ECG classification framework

The ECGDL framework consists of two main sets of properties. The first one is the open-source codebase, together with an arrhythmia label dictionary, which enable setting up experiments across datasets and classification tasks. The second set are categorizations and guidelines to drive experimental setup decisions.

### Open source codebase

#### Framework Implementation

Multiple datasets, segmentation and classification methods, as well as evaluation alternatives, were implemented as an object-oriented framework—ECGDL in the Python programming language. The base classes in ECGDL are: *Dataset*, *Classifier*— classification model—and *Transform*—segmentation technique, with the most important component being the *Experiment* class.

An *Experiment* instance is defined by a *Dataset* instance, a *Classifier* instance and a *Transform* instance, as well as additional attributes, most important of which are the evaluation paradigm and classification task. The *Experiment* class contains the main methods in the framework; namely, the classification training pipeline is executed in the *run* method, while the *evaluate* method calculates the evaluation metrics on the test set.

The framework serves two main purposes. The first is to implement the functionalities necessary to execute comparative studies of arrhythmia classification, like the one presented in this paper. These experiments are limited to certain classification tasks, as well as combinations of chosen neural networks and segmentation methods. The second purpose of the framework is to provide an extendable ECG classification codebase, with configurable components. ECGDL can be extended by adding new datasets and neural network architectures, as well as new parameter settings. Even with the components presented in this papers, in terms of datasets, segmentations and deep models, many more combinations could be investigated, which are out of the scope of this paper. In order to serve its extensibility purpose, the codebase of the framework has been made publicly available through GitHub as an open-source project.

#### Label dictionary for defining custom tasks

The machine learning task in this framework is classifying ECG segments into different types of normal and abnormal beats (arrhythmia). The framework enables the definition of various custom arrhythmia classification tasks as sets of target classes, which can be done by the user in a configuration file in comma-separated values (.csv) format. This is enabled by the label dictionary we devised, which is a file that unifies arrhythmia annotations across data sources, by mapping each distinct label in the dataset to the arrhythmia type it symbolizes. For example, atrial fibrillation is denoted by AFIB in some, and by AF in other datasets, and this is unified by the dictionary. Furthermore, ECG classification was set up as the base task, which means that the framework could potentially be extended to other classification tasks by providing a label mapping dictionary for the new end-task family and implementing new datasets as *Dataset* subclasses.

**Figure 1:**
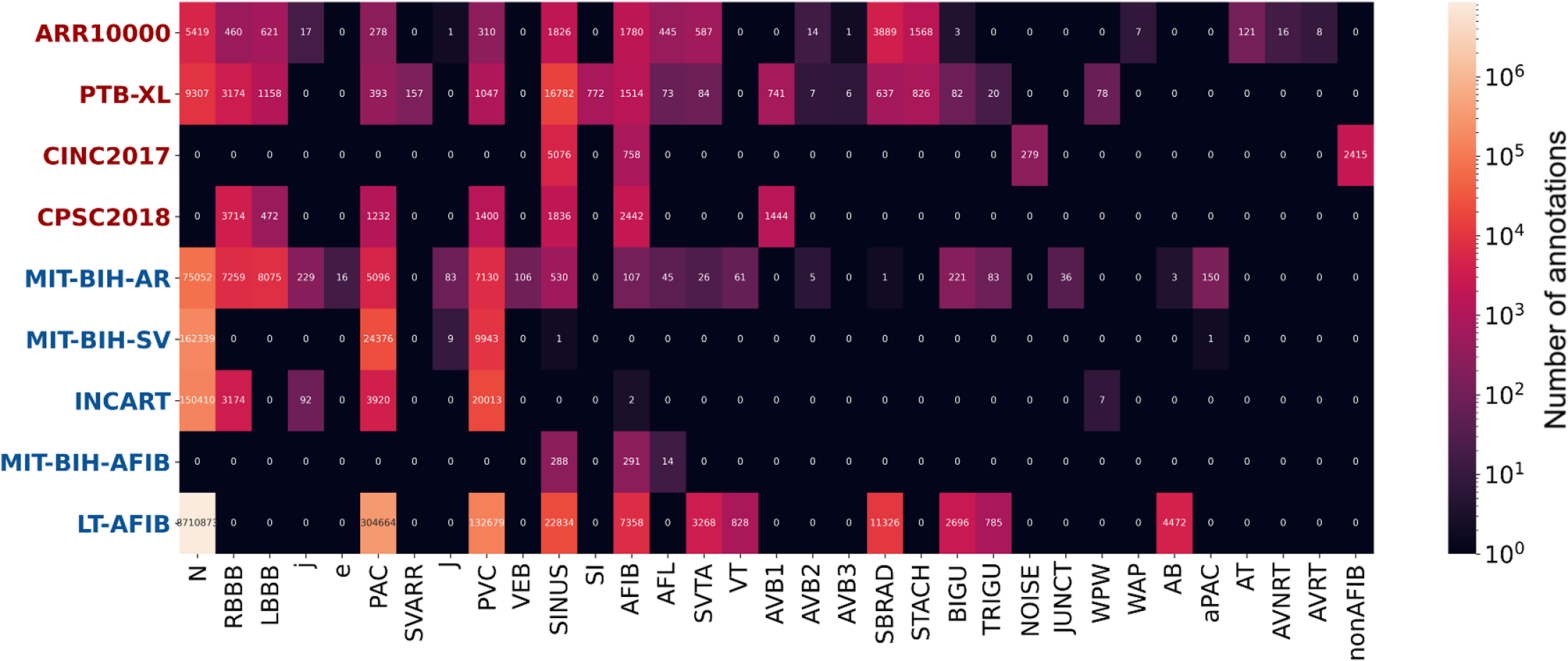
Label distribution in each dataset.

The arrhythmia types included in the label dictionary were selected based on their prevalence, clinical importance, and common usage in research. In addition, we wanted to include the most common types of heartbeat form abnormalities (corresponding to a single heartbeat), as well as rhythm abnormalities (spanning at least a few heartbeats). The choice of the datasets also considered the arrhythmia types they contain, as well as their wide use in the research field and their size. The distribution of different types of arrhythmia labels, for each dataset, is given in Fig. 1. In this figure, we can observe that premature ventricular (PVC) and atrial beats (PAC) are present in most datasets, as well as atrial fibrillation (AFIB), which are also the most significant types of arrhythmia according to relevant guidelines^31,32^. In addition, we can see that generally the datasets MIT-BIH-AR, PTB-XL and ARR10000 have the largest variety of arrhythmia labels. It should be noted that this figure presents the number of distinct annotated occurrences of each arrhythmia type; however, due to the different annotation types, these numbers are not directly comparable when considering total ECG duration or final number of training samples.

### Categorizations and guidelines

This section will present a categorization of ECG datasets, and guidelines related to segmentation and evaluation, which can be used to drive decisions when setting up experiments for arrhythmia classification.

#### Dataset categorization

One of the main tasks in the ECGDL framework is to unify the processing of ECG from different data sources, namely the most common publicly available datasets. One of the most significant features of each dataset to be considered is the way diagnostic information is represented in them. We have identified two main types of annotations: beat-level and recording-level, which are also the two dataset categories. Beat-level annotations provide a label for each heartbeat, which also includes information about the exact location of the R-peak. These annotations are common for the datasets in the PhysioNet repository^18^. The names of these datasets are highlighted with bold italic throughout this paper. PhysioNet beat-level annotations include two types: heartbeat annotations, which describe a single beat; and rhythm annotations, which point to the locations where specific rhythm episodes start and end. On the other hand, recording-level annotations are used to describe an entire ECG sequence with a single label, or a few labels in rare cases, disregarding the locations of specific beats. Recording-level annotated datasets are highlighted in bold throughout the paper.

#### Segmentation technique guidelines

ECG recordings, especially those in beat-level annotated datasets, require that the ECG sequence is split into shorter segments. There are various techniques to achieve this, which include heartbeat segmentation, episode segmentation and sliding window. Heartbeat segmentation allows for very precise and localized detection of abnormalities, however it is dependent on QRS detection algorithms. This adds additional complexity to the methods and QRS detection can be a challenging task in noisier recordings. Furthermore, episode segmentation for rhythm classification requires that the locations where the heart rhythm changes are known beforehand. This type of information is not available in raw continuous unannotated ECG signals, which is the most realistic use-case scenario of arrhythmia detection. Due to this, we propose the use of sliding window segmentation in beat-level datasets, for both rhythm and heartbeat form arrhythmia tasks, where windows have labels pertaining to their entire duration, without attaching them to a specific location. This is in line with recent research trends, such as large-scale arrhythmia studies with ECG sensors^9^, as well as recently published datasets^16^, because sliding window essentially transforms beat-level annotated datasets into recording-level ones. This type of segmentation is already being used for rhythm tasks, however it still needs to be confirmed that it can achieve satisfactory accuracy for heartbeat form tasks, which is one of the goals of our experiments.

#### Novel evaluation schema

Evaluation scenarios are an important topic in ECG classification, especially considering that datasets include many segments from the same patient and that arrhythmia classes are highly imbalanced. There exist different approaches to defining training and testing splits: intra-patient, holdout inter-patient, leave-one-patient-out and patient-specific evaluation (explained in the Methods section). With the advantages and disadvantages of existing evaluation schemas for ECG classification in mind^33,34^, as highlighted in the Methods section, we propose a novel schema, which we term **inter-patient cross-validation**. It adheres to the inter-patient principle by considering all segments from one patient as an undividable group and including them either in the train or in the test set. At the same time, cross-validation is performed by splitting the dataset into *k*-stratified folds of patients. More information about the optimization procedure which splits the dataset into stratified groups of patients can be found in the Methods section. In this way, compliance with the realistic inter-patient paradigm is achieved, as well as repeated validation enabled by multiple splits, which makes inter-patient cross-validation a good guideline for most datasets. More details about the implementation of this schema are given in the Methods section.

In cases with fewer distinct patients or whenever the arrhythmia task makes the selection of suitable stratified splits impossible, we suggest using the leave-one-patient-out evaluation scheme as an alternative option. In addition to inter-patient cross-validation, the ECGDL framework also implements intra-patient, leave-one-patient-out and patient-specific evaluation.

The schemas apply specifically to beat-level datasets, while in recording-level ones, each patient is represented with exactly one sample and the notion of inter- and intra-patient is lost. In this case, a simple cross-validation can be used. Some datasets, such as PTB-XL, already provide predefined cross-validation splits, taking into account the distributions of ECG classes in each split (these have also been included in the framework implementation).

## Experiments and results

In this section, we describe how we used the proposed framework to define and carry out experiments for arrhythmia classification using ECG, including the tasks and the datasets, the classification and segmentation settings, as well as the evaluation setup. Afterwards, we present the results from the comparative analysis on the form and rhythm tasks.

### Experimental setup

#### Tasks and datasets

Based on the analysis of related public challenges and notable arrhythmia detection studies, we cover the most significant arrhythmia types by grouping them into two tasks: rhythm and form.

**Figure 2:**
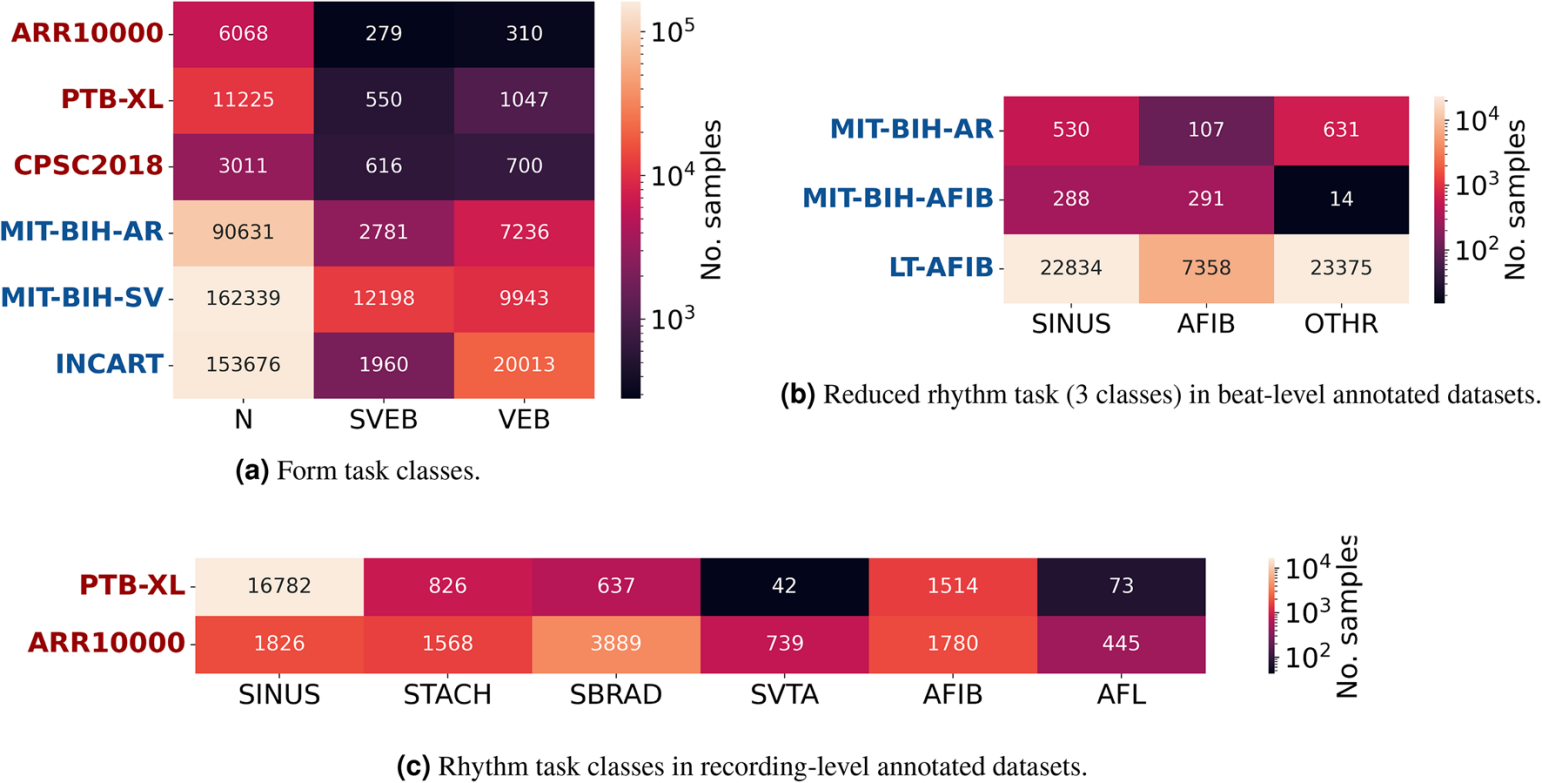
Distribution of classes for each task in different datasets.

The form classification task consists of the following classes: normal (N), supraventricular (SVEB) and ventricular beats (VEB), following the guidelines by the Association for Advancement of Medical Instruments (AAMI)^31^, and the class distributions for each dataset are given in Fig. 2a.

The class distribution of the rhythm task is given in Fig. 2c for the recording-level ARR10000 and PTB-XL datasets. Included classes are normal sinus rhythm (SINUS), sinus tachycardia (STACH) and bradycardia (SBRAD), as well as supraventricular tachycardia (SVTA) and atrial fibrillation (AFIB) and flutter (AFL). We can see that PTB-XL has a greater rhythm class imbalance than ARR10000. Even though PTB-XL has a larger number of samples overall, over 15,000 of those are SINUS segments, while SVTA and AFL have only around 100 samples. ARR10000, on the other hand, includes a sufficient number of samples from all classes. The most significant and common rhythm abnormality, which a lot of arrhythmia detection works attempt to distinguish it, is AFIB. Since some of the datasets do not include enough samples of the other rhythm types, we evaluate on an additional reduced rhythm task focusing on distinguishing AFIB from SINUS rhythm and other rhythms. The distribution of these three classes in the beat-level datasets is given in Fig. 2b. It should be noted here that this figure shows the number of episodes, while the number of training segments depends on the segmentation type and is generally larger than the number of distinct episodes. Important to note here is also that the LT-AFIB dataset is significantly larger than MIT-BIH-AFIB and MIT-BIH-AR, with over 20 times more samples in each class.

#### Classification and segmentation settings

An overview of the segmentation settings is given in Table 1. There are three segmentation techniques: heartbeat (*beats*), sliding window (*windows*) and full sequence input - no segmentation (*sequence*).

Each segmentation setting is denoted with the name of the technique and the segment duration in seconds. We consider two types of heartbeat segmentation, *beats0.72s* and *beats2.4s*. In the first type, *beats0.72s*, each generated segment corresponds roughly to a duration of one heartbeat^35^. The second type, *beats2.4s*, corresponds^11^ to a heartbeat and its two neighboring ones. Furthermore, we include two types of sliding window segmentation for beat-level datasets, where *windows2.5s* was chosen as similar in duration to *beats2.4s*, as well as to match *windows2.5s* segmentation on recording-level datasets^30^. The longer sliding window variant, *windows10s*, was chosen to match the common ECG duration in the ARR10000 and PTB-XL databases. Additionally, other studies^36^ also use 10-s long segments from beat-level datasets. The third type of input generation technique is *sequence*, which does not segment the ECG. We have included one main option, *sequence10s*, since it matches recording-level datasets that contain recordings of 10 s^10^ or longer.

An overview of the deep learning approaches used in our experiments is given in Table 2. The table shows the original setting for each architecture, including the segmentation technique, the arrhythmia task, the dataset and the reference study. The input signal frequency is also given in Table 2. Our frequency pre-analysis showed that a change in the input sampling frequency does not significantly influence the classification results in the range between 250 and 500 Hz. Therefore, for the experiments in this paper, we keep the original input frequency for each model same as in the work where it was proposed.

The first method is a simple CNN architecture, proposed^35^ for beat form classification using the MIT-BIH-AR dataset. The second deep learning approach is the ResNet architecture, according to the implementation by Weimann^37^, where it was applied to the CINC2017 dataset. Similar ResNet variants have been used in related studies, such as by Sellami^11^ in combination with heartbeat segmentation with neighborhood, and by Strodthoff^30^ in combination with sliding window of 2.5 s. The third method included in the framework is the winning one^38^ from the China Physiological Signal Challenge in 2018, using the architecture we refer to as GRUAttNet. It is designed for 12-lead ECG data, however it can be modified to work with a single-lead ECG as well. The last method we consider is the RTA-CNN architecture, proposed by Gao^39^ for AFIB detection, using single-lead ECG. We trained each of these networks anew on each dataset, without utilizing pretrained models. In addition, as previously mentioned, we use the pre-defined network settings without hyperparameter tuning for each experimental scenario.

**Table 1:** Overview of the segmentation techniques used in the experiments, with the chosen segment durations and reference studies.

Segmentation	Segment duration (s)	Notation
*beats*	0.72 s^35^	*beats0.72s*
	2.4 s^11^	*beats2.4s*
*windows* (beat-level)	2.5 s	*windows2.5s*
	10 s^36^	*windows10s*
*windows* (recording-level)	2.5 s^30^	*windows2.5s*
*sequence*	10 s^10^	*sequence10s*

**Table 2:** Overview of the four architectures included in the framework and the characteristics of the original pipelines in related studies where they were proposed.

Architecture	CNN	ResNet	GRUAttNet	RTA-CNN
Segmentation	*beats*	*sequence*, *beats* and *windows*	*sequence*	*sequence*
Dataset	MIT-BIH-AR	CINC2017, MIT-BIH-AR, PTB-XL.	CPSC2018	CINC2017
References	^35^	^11,30,37^	^38^	^39^
Frequency (Hz)	360	250	500	300

#### Evaluation setup

According to the evaluation guidelines developed within the framework, we use the inter-patient cross-validation scheme in all experiments. The dataset is split into 10 folds, where each group serves exactly once as a test set. In addition, one of the other folds is used as a validation set during training (for early stopping) and the test set is used for the calculation of performance metrics exclusively. We compare the methods with macro F1 score, which is a standard metric for imbalanced classification, including arrhythmia detection^9,10,40,41^. The use of macro-averaging (each class has equal weight) allows for a more realistic estimate of the performance due to the high class imbalance, where other variants such as micro-F1 would present overly optimistic numbers. The presented scores are averages over the 10 folds.

### Form classification results

**Figure 3:**
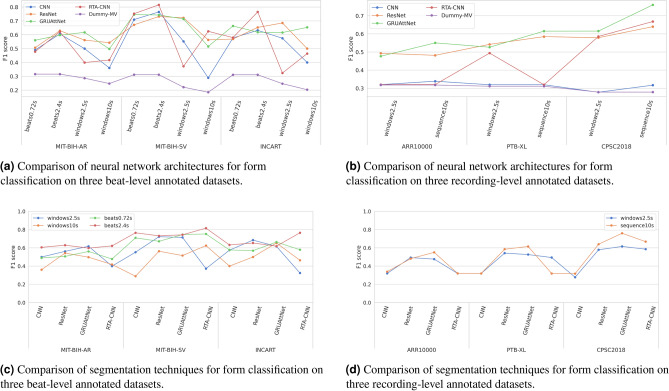
Results of the form classification experiments.

We will discuss the results from two perspectives: comparing model architectures and comparing segmentations. A comparison of the four architectures in terms of F1 scores is given in Fig. 3a for beat-level datasets and in Fig. 3b for recording-level datasets. We also show the scores for a dummy classifier which always predicts the majority class (Dummy-MV). In Fig. 3a, we can see that the CNN architecture exhibits a weaker performance than the remaining three. RTA-CNN results in notably high scores in some settings, such as with *beats2.4s* on MIT-BIH-SV and INCART, however when combined with sliding window, e.g. *windows2.5s*, its performance drops drastically in all three datasets. Similar observations regarding CNN and RTA-CNN can be made from the experiments on recording-level datasets shown in Fig. 3b. CNN is notably weaker that the other architectures, while RTA-CNN results in inconsistent scores in different settings. CNN, and RTA-CNN in some cases, is not better than a random classifier, suggesting it did not learn anything useful from the available data. ResNet and GRUAttNet perform similarly across all datasets and segmentations examined, as can be seen in both Fig. 3a,b. Both result in more consistent scores across different segmentations, as opposed to CNN and RTA-CNN. Still, GRUAttNet stands out as better performing than ResNet in both groups of datasets and as the best overall across the recording-level datasets.

Next, we analyse the same scores by comparing different types of segmentation. The scores are graphically presented for easier comparison in Fig. 3c for beat-level datasets and in Fig. 3d for recording-level datasets. On the three beat-level annotated datasets, two types of segmentation have been examined: heartbeat segmentation (both *beats0.72s* and *beats2.4s*) and sliding window (*windows2.5s* and *windows10s*). The results in Fig. 3c show that *beats2.4s* performs the best overall in terms of macro F1, outperforming both *beats0.72s* and the sliding window variants (*windows2.5s* and *windows10s*). When using the RTA-CNN architecture, and to a lesser extent the CNN architecture, we can see that beat segmentation techniques consistently outperform sliding window techniques across all datasets. However, with ResNet and GRUAttNet, the scores of different segmentation techniques are much closer, with *beats2.4s* and *windows2.5s* achieving similar classification performance and slightly outperforming the other two techniques. In addition, we can conclude that segmentation techniques with segment lengths corresponding to a few heartbeats perform the best, while longer (*windows10s*) and shorter segments (close to one heartbeat length), such as *beats0.72s*, result in lower classification scores.

As shown in Fig. 2a, recording-level datasets suitable for the form task are: ARR10000, PTB-XL, and CPSC2018. Classifying heartbeats according to form into the AAMI classes on these three datasets is a challenging task because heartbeat form is commonly classified with beat-level processing of ECG. To perform form classification on such recordings, where beat annotations are not available, we experiment with two input variants: full sequence input (without segmentation) and sliding window. Sliding window enables the analysis of ECG on a smaller scale but also requires that the predicted labels for each window are aggregated to obtain a single prediction. The results are summarized considering F1 score in Fig. 3d. Here we included *sequence10s* and *windows2.5s* as the two segmentation variations. Full sequence input outperforms sliding window segmentation in most cases on all datasets. This could indicate that the error introduced by assigning the recording label to each of the windows outweighs the potential benefits of smaller-scale processing with windows.

### Rhythm classification results

**Figure 4:**
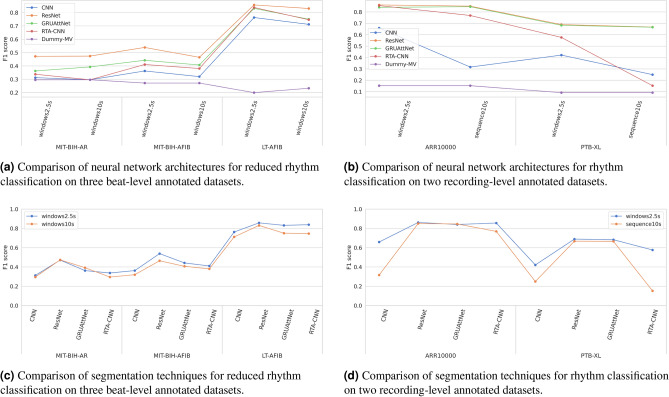
Results of the rhythm classification experiments.

A comparison of the neural network architectures in terms of F1 scores is given in Fig. 4b for recording-level datasets on the rhythm task and in Fig. 4a for beat-level datasets on the reduced rhythm task. In addition, the F1-scores from a dummy majority vote classifier are also shown. On the three-class reduced rhythm task, Fig. 4a clearly shows that ResNet outperforms the other three architectures across all datasets and segmentations. CNN is again the weakest-performing, with RTA-CNN and GRUAttNet falling in the middle. The four architectures achieve consistent scores relative to one another in all settings. However, we can observe that for MIT-BIH-AR, both CNN and RTA-CNN are unable to beat a random classifier, suggesting that they were not able to learn meaningful patterns in the data, which is not the case for LT-AFIB. On the six-class rhythm task presented in Fig. 4b, both ResNet and GRUAttNet work well with very close scores on both datasets and segmentations.

The two types of segmentations analyzed for rhythm classification: *windows2.5s* and either *windows10s* or *sequence10s*, depending on the dataset type, are graphically compared in Fig. 4c for beat-level datasets, and in Fig. 4d for recording-level datasets. From both figures we can observe that, depending on the dataset type, *windows2.5s* works better than the other segmentation technique with longer segment lengths (*windows10s* and *sequence10s*).

### Classification results on original tasks for each dataset

In addition to the comparative analysis on two general tasks - form and rhythm, we used the ECGDL framework to perform experiments on the original tasks specific for some of the datasets, which allows for comparison with related studies. The results from this analysis can be found in one of our previous works^42^. The most notable conclusion from the study is that on both tasks on the ARR10000 database (rhythm task and reduced rhythm task), as well as for rhythm classification on PTB-XL, our state-of-the-art methods, chosen due to their performance on other datasets and tasks, achieve higher scores than those previously reported in literature. This proves that the comparative analyses made possible with ECGDL are beneficial for the advancement of the field of computational ECG analysis.

### Additional results

Additional results from the analysis using the ECGDL framework can be found in the ECDGL github repository, or more specifically at the following link: https://github.com/elenamer/ecg_classification_DL/tree/main/results_tables. These additional results include: inter- vs. intra-patient cross-validation evaluation for form classification on three beat-level datasets; train, test and validation scores for form and rhythm classification on both beat-level and recording-level datasets; and per-class metrics. Moreover, results for patient-specific models on data acquired from a mobile ECG device can be found in one of our previous works^43^ .

## Discussion

The main contribution in this paper is the public ECG classification codebase - ECGDL, one of the first efforts that includes 9 arrhythmia datasets, covering a large number of both morphological and rhythmic arrhythmia types, as well as 4 deep learning models, 4 segmentation techniques and 4 distinct evaluation schemes. A comparative analysis along each of these framework components has provided a unique comprehensive perspective into arrhythmia classification. Moreover, the framework is flexible and configurable to be easily extended with additional datasets and methods. Therefore, it is a significant contribution towards more standardized evaluation of ECG classification methods.

In order to be able to define general arrhythmia classification tasks, the class representation in each dataset is unified by creating a class mapping dictionary: to map the dataset-specific labels to common arrhythmia categories. This enables the definition of custom tasks with sets of target classes that are general and common for all datasets included. We extend the evaluation of beat-level annotated datasets on rhythm tasks by applying sliding window segmentation, achieving comparable scores to when the standard segmentation on beat-level annotated datasets is used, i.e heartbeat segmentaion. Considering the added complexity in heartbeat segmentation (it requires prior QRS detection), sliding window shows to be a promising direction for future ECG-based arrhythmia classification under one umbrella of segmentation. A sliding window segmentation with a segment of 2.5 s (duration close to 3 normal heartbeats) was found as the best solution. In the form experiments, on beat-level annotated datasets, a similar conclusion can be made: heartbeat segmentation with a segment duration close to 3 heartbeats is the best option. For form classification on recording-level annotated datasets on the other hand, full sequence input of 10 s is the best-performing technique.

In the experiments, when comparing the models, we can see that GRUAttNet and ResNet generally outperform the other models across multiple datasets and tasks. GRUAttNet and ResNet have similar training times in most settings and achieved similar F1 scores in rhythm classification, while for form classification, GRUAttNet slightly outperforms ResNet. This could be explained by the fact that the architecture of GRUAttNet has the largest variety of mechanisms, utilizing both GRUs and attention, in addition to convolutional layers. Another reason could be that the CPSC2018 task, for which this model was developed, included both form and rhythm labels, making this model the most general.

A component of high significance is the evaluation schema. A fair and realistic evaluation procedure would ensure stratified sampling and cross-validation and would follow the inter-patient paradigm. Considering these parameters, we propose the novel cross-validation inter-patient evaluation scheme as the best option overall. In addition, in line with related studies, we highlight the need to always use metrics suitable for imbalanced classification, such as macro F1 score, in order to be able to compare results from different studies. Our experiments show that form classification is a more challenging task, according to the scores obtained. Regarding rhythm classification, the results show that the best methods are capable of successfully classifying atrial fibrillation segments with high precision and recall scores.

Additionally, we perform benchmarking on a set of state-of-the-art tasks, related to each of the datasets. These tasks differ from the general form and rhythm tasks because they can only be applied to one dataset. Using only a single ECG lead, we are able to come close to most state-of-the-art reported results, with the exception of the PTB-XL form benchmark. In addition, we are able to improve the reported scores on the two ARR1000 benchmark tasks, as well as on the PTB-XL rhythm task, achieving a higher F1 score than the current best result.

The analyses in this paper have a few limitations, namely the lack of hyperparameter tuning and limited configurability of the neural networks used, as well as the fixed ECG lead positioning. The ECGDL framework could easily be extended for future works exploring effects of different leads, as well as to other ECG analysis tasks, including multi-label classification and multi-modal approaches combining other physiological signals, as well as transfer learning scenarios.

## Methods

### Implementation

This section will provide more details about the implementation of the ECGDL codebase. We chose to use the Keras library^44^ with TensorFlow^45^ backend. Additionally, we also make use of the scikit-learn^46^ library, the waveform-database (WFDB)^47^ Python package, the SciPy library^48^, as well as the NumPy library^49^. Furthermore, we utilized the Matplotlib^50^ and the Seaborn^51^ visualization libraries. In order to track the neural network experiments, we also utilize the Weights and Biases (WandB)^52^ platform with its corresponding Python package, however this dependency is not crucial for the functioning of the arrhythmia detection framework.

**Figure 5:**
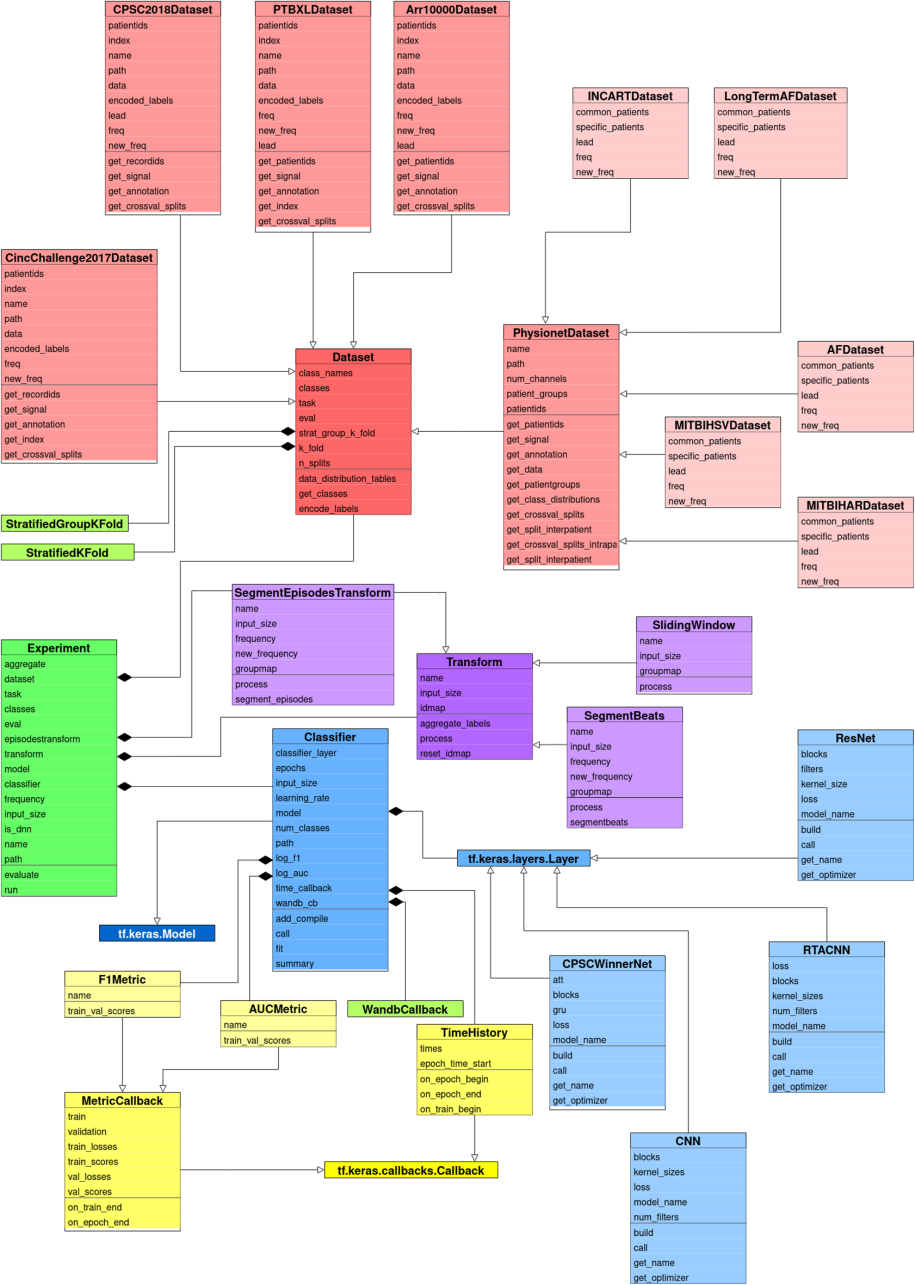
UML diagram of the classes that comprise the ECGDL arrhythmia detection software framework.

In order to obtain a clear overview of the framework’s structure, we provide the Unified Modeling Language (UML) class diagram in Fig. 5. The main base classes in the ECGDL framework are: *Dataset*, *Classifier* and *Transform*. The base *Dataset* class contains the label dictionary that maps classes to dataset-specific labels, as well as class distribution visualization functions. The most significant methods that each *Dataset* subclass implements are get_signal and get_annotation, as well as get_crossval_splits functions for both intra- and inter-patient evaluation. There is a *PhysionetDataset* class, which inherits from the base *Dataset* class, and serves as a base class to all datasets from the Physionet repository (excluding PTBXL due to its different labelling format). All Physionet datasets are characterized with the WFDB signal format and beat-level annotations, which makes their processing the same for the most part and can be implemented in the common *PhysionetDataset* class. Thereafter, the *PhysionetDataset* subclasses only contain dataset-specific attributes such as frequency and lead configuration, and fixed train-test splits for holdout inter-patient evaluation.

The different types of segmentation are implemented as separate classes that inherit from the *Transform* class. Each *Transform* subclass implements the process method, which for given ECG recordings and annotations, returns ECG segments with corresponding class labels. These ECG segments could be beats or windows of some predefined length. Additionally, label aggregation options are implemented in some of these *Transform* classes (if we want to calculate classification metrics at a level higher than window-level, such as recording-level). It is also worth mentioning that some *Transform* subclasses can only be used for a specific dataset type, such as *SegmentBeats*, which only works with *PhysionetDataset* instances.

Finally, the *Classifier* class inherits from the Keras *Model* class. It contains most training parameters, such as the number of epochs, learning rate, metric callbacks and one dense layer that outputs the predicted class probabilities. The rest of the neural network architecture is defined in separate classes that inherit from Keras’ *Layer*. Each *Classifier* instance contains such a network architecture variable. The network architectures presented in this paper are static, which means most of the architecture parameters, such as number of layers, as well as some training parameters, such as the optimizer and loss functions, are not configurable parameters. We wanted to limit the scope of this paper to fixed network settings, which have been shown to be the best in some ECG arrhythmia scenarios.

### Datasets

An overview of the datasets included in the framework and used in the experiments is given in Table 3. The table contains information about dataset size, in terms of number of recordings, and the recording length. We can conclude that in recording-level datasets, the ECG examples are much shorter, usually around 10 s and up to 1 min, when compared to the datasets with beat-level annotations. In addition, recording-level datasets are aquired from a significantly larger number of people. Another observation is that recording-level annotations are more commonly found in newer datasets, where both PTB-XL and ARR10000 were published in 2020, while the MIT-BIH datasets originate more than 20 years ago.

Among these nine datasets, different ECG measurement formats are included, ranging from the standard 12-lead resting ECG to monitoring with the Holter monitor using two ECG channels. Since the focus of this paper is analysis of methods applicable to single-lead ECG, we utilize one lead in 12-lead datasets. The modified limb lead II in the MIT-BIH-AR dataset has been extensively used in literature for arrhythmia detection from single lead ECG^36,53^; consequently, we choose limb lead II when 12-lead ECG is available in the dataset.

**Table 3:** Overview of the ECG datasets and categorization according to annotation type. ECG1 signifies a non-standard lead position.

Annotation type	Dataset name	Leads (selected)	Number of people/recordings	Recording duration
beat-level	MIT-BIH-AR^15^	2 (MLII)	47	30 min
	MIT-BIH-AFIB^54^	2 (ECG1)	24	10 h
	MIT-BIH-SV^55^	2 (ECG1)	78	30 min
	INCART^13^	12 (lead II)	75/32	30 min
	LT-AFIB^17^	2 (ECG1)	84	24 h
recording-level	ARR1000^16^	12 (lead II)	10,646	10 s
	PTB-XL^14^	12 (lead II)	18,885/21,837	10 s
	CPSC2018^41^	12 (lead II)	6877	6–60 s
	CINC2017^40^	1 (MLI)	8528	9–60 s

#### Ethics statement and consent to participate

The datasets used in the study are open access and have been previously published, as indicated with the references for each dataset in Table 3. Therefore, no ethics statement and informed consent is required for this study. Data processing was performed in accordance with relevant guidelines and regulations, and the study was carried out in compliance with the Declaration of Helsinki.

### Data preparation

This framework focuses on deep learning pipelines for ECG classification. Neural networks are themselves able to extract meaningful features and learn to classify ECG segments, which is why no significant preprocessing of the ECG signals is needed. However, the data still needs to be modified to a format suitable to serve as neural network input, which requires a uniform input length. ECG signals sometimes come in varying durations, which means they need to be either lengthened or shortened. We do this by either simple cropping of the signal or zero-padding, as commonly done.

The neural network architectures that we chose to include in the experiments have been proposed for different datasets, which means that they are aimed at specific signal sampling frequencies. The framework allows for the network input frequencies to be customized; however, the experiments in this paper are limited to pre-defined network architectures, without hyperparameter tuning, where each network architecture is tied to a specific frequency. We performed a preliminary analysis for different input frequencies to confirm that the frequency does not have a significant impact on the arrhythmia classification results. To achieve this, as part of the data preparation, we also perform resampling of input signals to the frequency tied to the network. Each of the datasets given in Table 3 has a different original frequency, however during the data preparation procedure, they are resampled to the input frequency of the network. Furthermore, the lengths of the segments in different types of segmentation are defined in seconds, which means that with different frequencies, the number of samples in a segment will vary. The data preparation described in this section is necessary both when full recordings in recording-level datasets are used, referred to as *sequence* segmentation in the results, and in combination with segmentation techniques.

### Segmentation

In order to process a continuous ECG, current state-of-the-art methods split the sequence into smaller segments. This step of the pipeline is called segmentation. The segmentation method depends heavily on the format in which the dataset is given, namely beat-level annotations or recording-level annotations. In the following sections, the main types of segmentation will be presented. It should be noted that heartbeat segmentation and episode segmentation can be used exclusively on datasets with beat-level annotations.

#### Heartbeat segmentation

**Figure 6:**
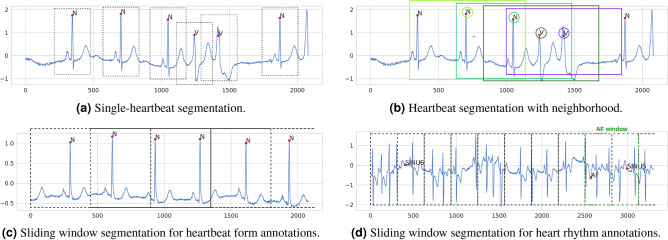
Segmentation techniques for beat-level annotated datasets.

A large portion of arrhythmia detection research performs classification on a beat-by-beat level. This enables a precise localization of heartbeat form abnormalities. Heartbeat segmentation is only possible for beat-level annotated datasets, where exact heartbeat locations are determined by the location of the heartbeat’s R-peak, which requires prior QRS detection. This type of segmentation can be implemented in different ways. Static segmentation is common, shown in Fig. 6a, where a window of certain length (usually around 0.8 s, which is the common heartbeat duration^56^), centered around the R-peak, represents one heartbeat. Another option are so-called dynamic segmentation variations, where the heartbeat window is not centered, but its start is determined by other characteristic ECG waves. In ECGDL, we only include static segmentation.

In addition to single-beat segmentation, the neighbouring beats have also been shown to be helpful for beat classification^11^. We refer to this type of segmentation as heartbeat segmentation with neighborhood and it includes an extended window around the heartbeat we want to classify, which also covers its two neighboring beats, as displayed in Fig. 6b. It should still be noted that, for this variation, R-peak locations are also significant, since we focus on classifying a specific beat and the segment is aligned according to the R-peak location. Heartbeat segmentation variants are denoted by *beats* in the experimental results, where heartbeat segmentation with neighborhood is implemented by choosing a longer segment duration.

#### Episode segmentation

The second type of segmentation that can be used for datasets with beat-level annotations is episode segmentation. In addition to single-heartbeat abnormality annotations, these datasets also contain rhythm information, with annotated start and end of each distinct rhythm episode. Each of these distinct rhythm episodes can be segmented and classified separately^36^. This type of segmentation has some practical implications, which make it unsuitable for fair evaluation. Firstly, some arrhythmia types are naturally shorter than others on average. If each such episode from start to finish is a separate segment to classify, its duration could leak some information about the class label. In addition, without annotations, it is impossible to know where the rhythm changes. So in order to perform this type of segmentation, rhythm change locations are necessary. This type of information is not available in raw continuous unannotated ECG signals, which is the real-life use-case scenario of arrhythmia detection methods. Due to this, we do not include this segmentation in our experiments.

#### Sliding window segmentation

The third main type of ECG segmentation are sliding window techniques, where a longer ECG signal is split into smaller segments of predefined duration, disregarding the heartbeat locations. The windows are also often overlapping, usually with a 50% overlap in inter-patient evaluations, while with intra-patient evaluations, no overlap between windows is introduced to avoid label leakage. This type of segmentation only needs the raw signals and no additional information, and segments are always formed from consecutive signal samples. Variations of the sliding window technique can be used both on beat-level annotated and recording-level annotated datasets. Beat-level annotated datasets usually contain long recordings (e.g. 30 min in MIT-BIH-AR^15^) and they can be transformed into a set of few-second long ECG segments using sliding window. Each of these windows usually contains several consecutive ECG beats, sometimes each with a beat form label, and it can also contain a rhythm change within.

One of the most important parts of sliding window segmentation is determining a label for the entire window. This label is inferred from all heartbeat form and rhythm annotations that pertain to the part of the signal falling withing the window. One option is to label the segment with all distinct labels appearing in that window (needs to be addressed with multi-label classification), while another one is to choose the label that pertains to the largest portion of the signal or appears most frequently (also known as voting)^36^. In ECGDL, sliding window is implemented by assigning a single label to each window using voting. For heartbeat form classes, often the majority of the beats are normal and the goal is to recognize abnormal beats. To this end, in ECGDL, if at least one heartbeat is labeled as one of the abnormal classes, the window is assigned the abnormal class label, even if all other beats in the window are normal. An example in the case of beat form labels is shown in Fig. 6c, while for rhythm labels in Fig. 6d.

Regarding recording-level annotated datasets, determining the ground truth label is more straightforward. In that case, each recording (generally shorter: between 10 s and a few minutes) is split into smaller windows, each with the same label as the one assigned to the full recording. Each of these windows is then processed through the classification pipeline separately, and is assigned an independent class during the prediction stage. After the predictions for each window are obtained, a single class prediction needs to be determined for the entire recording. This is commonly done by majority voting^30^.

### Evaluation schemas

The evaluation setup for supervised learning tasks on ECG signals must carefully consider how the training and testing data are split. This mainly concerns beat-level annotated datasets, where segmentation of longer ECGs is needed. A key distinction is whether the testing set includes ECG segments from patients that were also included in the training set (intra-patient paradigm) or whether the testing set only includes ECG segments from new, unseen patients (inter-patient paradigm)^31^. In addition, there is also patient-specific evaluation^43^, where personalized models for each patient are trained and including limited ECG segments from the patient in question in the training set is allowed^2^.

The inter-patient paradigm is commonly used as a simple holdout validation^11,12,57,58^— holdout inter-patient evaluation. For example, the MIT-BIH-AR dataset is split into two predefined groups of patients, where one serves as train and the other as a test set^59,60^. The inter-patient paradigm is the most realistic one, however the already established static splits could imply low utilization of the data available (the test set of MIT-BIH-AR is 50% of the data), and skewed data distribution which does not generalize well to real scenarios^6^. To mitigate some of these issues, some more recent studies propose leave-one-patient-out evaluation^33^, where the inter-patient paradigm is combined with cross-validations and each patient serves as a test set exactly once. This schema is realistic and allows estimation of the stability and generalizability of the models, however with large number of patients, it could become unfeasible since many models need to be trained. The intra-patient paradigm, on another hand, can also be implemented as cross-validation—intra-patient cross-validation - with an arbitrary number of folds, where train and test sets are random splits of all segments, disregarding which patient they originate from. This is a biased heartbeat selection^34^ and does not translate well to realistic scenarios, however it is still used by a large portion of research^35,36^.

As an alternative to these schemas, we propose using inter-patient cross-validation. The schema is a cross-validation schema on the inter-subject level, implementing the realistic inter-patient paradigm by always including all beats from one patient either in the train or in the test set. This is achieved by considering all segments from one patient as one indivisible group and splitting the dataset into k folds of patients (groups of segments). These folds are used for cross-validation. The main challenge with this type of evaluation is finding a k-fold split of the groups while keeping the split stratified, since usually each patient has a heavily imbalanced class distribution. To split the dataset into stratified groups, we propose using an optimization method that attempts to reduce the mean standard deviation among the class distributions in the different splits. In the framework, this is implemented using a method from the scikit-learn^46^ Python library, called *StratifiedGroupKFoldSplit*, and it can be applied to find the stratified split of any beat-level dataset. This method iterates over all groups and assigns the group to the most suitable fold (at that moment) according to a heuristic, which in our case is the mean (over all folds) standard deviation of the number of samples of each class. This way suboptimal splits in terms of stratification will be produced, however for the purpose of this evaluation, this method provides the needed functionality.

### Neural network architectures

We chose to limit the learning algorithms in the framework to only deep-learning based methods, since they require minimal data preprocessing, are more generalizable and robust than other classifiers, and are simpler to transfer to multiple datasets. Another characteristic of deep neural networks is the need for large datasets in order to achieve high classification scores, which can be both an advantage and a disadvantage in different situations. The main disadvantages of these methods are the required large computational power to train them and the limited explainability of the trained models.

We include four neural network architectures in the ECGDL framework and they are presented in the following subsections, together with the implementation-specific parameters. They were chosen to be representative of the trends in arrhythmia classification literature, as well as to include a variety of deep learning techniques.

#### Convolutional neural network (CNN)

Convolutional neural networks (CNNs), which extract knowledge from the input using the convolution operation, are one of the most widely used types of neural networks^61^. Convolutional layers are the main building blocks of all types of CNNs. A convolutional layer aims to learn feature representations of the inputs by learning convolution kernels (filters) used to compute different feature maps^61^. When applied to time-series data, such as ECG, one-dimensional convolutional layers are commonly used^62^. These layers are used for feature extraction from raw ECG data in the CNN architecture described in this section, as well as in the other three architectures included in this framework, where they are combined with additional deep learning mechanisms.

The first architecture that we experiment with is a simple CNN model, which has been proposed by Acharya^35^ for heartbeat classification. It comprises three CNN blocks, where each block contains a single CNN layer, followed by a *max pooling* layer^61^ with stride 2. The purpose of *max pooling* is to reduce the size of the feature map output by the convolutional layers. The convolutional layers have 5, 10 and 20 filters and filter sizes of 3, 4 and 4, respectively. The convolutional blocks are followed by three fully-connected layers, with 30, 20 and N (number of classes) units, respectively. The first two fully connected layers and the convolutional layers all have leaky rectifier linear unit (*LeakyRelu*) activation^63^, while the last layer has *softmax* activation.

#### Residual network (ResNet)

The second network architecture we used is a residual network (ResNet). The residual learning paradigm^64^ reformulates the layers as learning residual functions with reference to the layer inputs. This is achieved by including shortcut connections, skipping one or more layers, which allows for untransformed input to directly be propagated to the output^61^. Residual networks are commonly organized in blocks of convolutional layers.

Different ResNet variations have been used in arrhythmia detection works^9,11,30,37^. The version included in this framework has been adapted from an ECG classification pipeline aimed at transfer learning^37^. The implementation consists of four blocks, with two convolutional layers in a block, each with filters of size 3. The number of filters in the layers in each block are: 64, 128, 256 and 256, respectively. Each block includes *batch normalization*^65^ and *dropout* regularization^66^ between the convolutions. Shortcut connections are added around each block in the form of projection shortcuts^64^. There is one *global average pooling* (GAP) layer at the end of the network, before the classification layer with *softmax* activation. When compared to the previous CNN, this is a larger network with a depth of a total of 9 convolutional layers, with a large number of units (filters) in each layer.

#### GRUAttNet

Convolutional layers extract features from ECG waveforms, however additional mechanisms can be used to capture temporal dependencies in signals. Several recurrent neural network (RNN) variations are most commonly used in practice, such as gated recurrent units (GRUs)^67^, which solve the vanishing gradient problem and enable capturing long-term dependencies^68^. Another important deep learning mechanism is attention. Attention mechanisms^69^ draw global dependencies between the input and the output by assigning weights to each part of the input sequence and this mechanism can be applied to the sequence outputs from an RNN^69^.

The third architecture that we use in our experiments includes both RNN with GRU units and attention. It was the winning model^38^ of the 2018 China Physiological Signal Challenge^41^ and we refer to it as GRUAttNet. It consists of five convolutional blocks, each containing three convolutional layers. The convolutional layers consist of 12 filters each, with filter lengths of 3, 3 and 24, respectively. Each layer is followed by a *LeakyReLU* activation and a *dropout* regularization layer at the end of each block. The blocks are followed by a bidirectional *RNN*^70^ of 12 GRU units and an *attention* layer. The outputs of the *attention* are used as input in the final fully-connected classification layer.

#### RTA-CNN

The fourth architecture used in this paper, RTA-CNN architecture with exponential nonlinearity loss (EN-loss), has been proposed for the CinC2017 challenge task and dataset by Gao^39^. This network is built from residual-based temporal attention blocks (RTA). Each block is composed of two branches: an attention branch to provide temporal attention weights, and a trunk branch for adaptively extracting and refining features according to the attention weights. In order to alleviate the class imbalance problem, the authors also propose a variation of cross-entropy loss called exponential nonlinearity (EN) loss.

This network consists of 6 RTA blocks, with the longest path through the block including 5 convolutional layers, totaling to 30 layers in depth. The blocks have 16, 32, 64, 64 and 128 filters in each convolutional layer, respectively. The convolutional layers in each of the 6 blocks have decreasing filter lengths; 32, 16, 9, 9, 3 and 3, respectively. This means that this network employs wider filter kernels than some of the other architectures. After the RTA blocks, there is a fully-connected layer with 100 units. Furthermore, *batch normalization* is used after each convolutional layer and *dropout* layers are added after the RTA blocks.

## Data Availability

Most of the datasets are available through the PhysioNet repository, which is an online platform for physiological and clinical data and related open-source software^18^. The majority of the ECG datasets on PhysioNet are given in the WaveForm DataBase (WFDB) format^47^, which has developed into a standard for the distribution of physiological signal data. In addition, ECG waveform datasets can be found on other public repositories such as figshare^19^, Zenodo^20^ and IEEE Data Port^21^. In order to serve its extensibility purpose, the codebase of the ECG framework has been made publicly available through GitHub as an open-source project at https://github.com/elenamer/ecg_classification_DL.
